# Expression and Antimicrobial Function of Beta-Defensin 1 in the Lower Urinary Tract 

**DOI:** 10.1371/journal.pone.0077714

**Published:** 2013-10-21

**Authors:** Brian Becknell, John David Spencer, Ashley R. Carpenter, Xi Chen, Aspinder Singh, Suzanne Ploeger, Jennifer Kline, Patrick Ellsworth, Birong Li, Ehrhardt Proksch, Andrew L. Schwaderer, David S. Hains, Sheryl S. Justice, Kirk M. McHugh

**Affiliations:** 1 Section of Nephrology, Nationwide Children’s Hospital, Columbus, Ohio, United States of America; 2 Center for Clinical and Translational Research, The Research Institute at Nationwide Children’s Hospital, Columbus, Ohio, United States of America; 3 Biomedical Sciences Graduate Program, The Ohio State University College of Medicine, Columbus, Ohio, United States of America; 4 Center for Molecular and Human Genetics, The Research Institute at Nationwide Children’s Hospital, Columbus, Ohio, United States of America; 5 The Ohio State University College of Medicine, Columbus, Ohio, United States of America; 6 Department of Internal Medicine and Pediatrics, University of Rochester, Rochester, New York, United States of America; 7 Center for Microbial Pathogenesis, The Research Institute at Nationwide Children’s Hospital, Columbus, Ohio, United States of America; 8 Department of Dermatology, University of Kiel, Kiel, Germany; Institut de Pharmacologie et de Biologie Structurale, France

## Abstract

Beta defensins (BDs) are cationic peptides with antimicrobial activity that defend epithelial surfaces including the skin, gastrointestinal, and respiratory tracts. However, BD expression and function in the urinary tract are incompletely characterized. The purpose of this study was to describe Beta Defensin-1 (BD-1) expression in the lower urinary tract, regulation by cystitis, and antimicrobial activity toward uropathogenic *Escherichia coli* (UPEC) *in vivo*. Human *DEFB1* and orthologous mouse *Defb1* mRNA are detectable in bladder and ureter homogenates, and human BD-1 protein localizes to the urothelium. To determine the relevance of BD-1 to lower urinary tract defense *in vivo*, we evaluated clearance of UPEC by *Defb1* knockout (*Defb1* ^*-/-*^) mice. At 6, 18, and 48 hours following transurethral UPEC inoculation, no significant differences were observed in bacterial burden in bladders or kidneys of *Defb1* ^*-/-*^ and wild type C57BL/6 mice. In wild type mice, bladder *Defb1* mRNA levels decreased as early as two hours post-infection and reached a nadir by six hours. RT-PCR profiling of BDs identified expression of *Defb3* and *Defb14* mRNA in murine bladder and ureter, which encode for mBD-3 and mBD-14 protein, respectively. MBD-14 protein expression was observed in bladder urothelium following UPEC infection, and both mBD-3 and mBD-14 displayed dose-dependent bactericidal activity toward UPEC *in vitro*. Thus, whereas mBD-1 deficiency does not alter bladder UPEC burden *in vivo*, we have identified mBD-3 and mBD-14 as potential mediators of mucosal immunity in the lower urinary tract.

## Introduction

The epithelial lining of the kidney and urinary tract is contiguous with the external environment via the urethra. Despite the anatomic proximity of the urethra to the skin, genital, and digestive tracts, urine generally lacks culturable bacteria and urinary tract infections (UTI) do not occur as frequently as might be expected. The properties of the urinary tract that promote sterility are not completely known. Proposed mechanisms include roles for regular bladder emptying, urothelium integrity, exfoliation of umbrella cells, elaboration of cytokines and chemokines that promote leukocyte recruitment and activation, mucus production, and synthesis of bactericidal and/or bacteriostatic peptides[1,2]. Antimicrobial peptides (AMPs) comprise a diverse group of molecules with bactericidal and immunomodulatory activity and may serve key roles in host defense against UTI. 

Defensins are one of the largest and most-studied families of AMPs in mammals. They are produced by a variety of epithelial and bone marrow derived cells and have broad-spectrum antimicrobial activity against gram-positive and gram-negative bacteria, viruses, fungi, and some protozoa[3–6]. Defensins are small cysteine rich proteins with a molecular weight of 3-5 kilodaltons consisting of a β-sheet structure linked by three disulfide bonds, and are classified into α- and β-defensins based on the pattern of disulfide bridges[6]. Over 28 β-defensin (*DEFB*) genes have been identified in humans[7], and expression has been localized to the kidney, skin, cornea, and mucosal epithelial cells lining the digestive, respiratory, and reproductive tracts[6]. The expression and biological relevance of β-defensins in the lower urinary tract have not been completely elucidated. 

Human β-defensin 1 peptide (HBD-1), encoded by the *DEFB1* locus, was initially isolated from the ultrafiltrate of patients undergoing chronic hemodialysis[8]. *DEFB1* mRNA expression is detected at high levels in the distal nephron and collecting system of the kidney[9]. *DEFB1* mRNA and HBD-1 peptide production were also observed in the distal ureter[10]. HBD-1 is synthesized as a 68 amino acid pro-peptide and undergoes variable amino-terminal processing to generate mature peptides ranging from 36 to 47 residues that are detectable in uninfected human urine[9,11]. Mature HBD-1 peptide levels are increased in patients with pyelonephritis[12]. The sensitivity of uropathogenic *Escherichia coli* (UPEC) to the bactericidal activity of HBD-1 peptide depends upon the length of the peptide as well as the presence of sodium chloride at physiological (micromolar) concentrations[9]. Given the variability in the length of HBD-1 peptides and sodium content of human urine, the potential protective capacity of HBD-1 in controlling UTI is unknown[9]. 

*Defb1* encodes murine BD-1 (mBD-1) and is orthologous to the human *DEFB1* gene[13–15]. As observed in humans, *Defb1* mRNA is detected in collecting ducts of adult kidneys[13–15]. Similar to the HBD-1 protein, recombinant mBD-1 protein exhibits salt-sensitive bactericidal activity *in vitro*, but its contribution toward clearance of uropathogens has not been tested *in vivo*[14–16]. *Defb1* knockout (*Defb1* ^*-/-*^) mice have a higher incidence of *Staphylococcus* spontaneous bacteriuria[16]. Although this observation is suggestive of a potential role in urinary tract defense, up to 70-80% of all UTI are caused by gram-negative organisms, particularly UPEC. In this study, we tested the hypothesis that *Defb1* is required for bacterial clearance of UPEC following transurethral challenge. 

## Methods

### Study approval and procurement of human tissue and urine

This study was approved by the Nationwide Children’s Hospital Institutional Review Board (IRB-07-00383). Human kidney, ureter, and bladder from pediatric patients without recurrent UTI were obtained from the NCH Department of Pathology as described[17]. 

### UTI mouse model

Maintenance of all mice was in strict accordance of the Institutional Animal Care and Use Committee (IACUC) rules and regulations. The mice had a normal 12- hour light-dark cycle and were maintained on standard chow diet (Harlan Laboratories, Indianapolis, IN). The experiments presented in this manuscript are approved (AR06-00119) by The Research Institute at Nationwide Children’s Hospital Institutional Laboratory Animal Care and Use Committee (Welfare Assurance Number A3544-01).Defb1^-/-^ mice on a pure C57BL/6 background were a kind gift of Dr. Lisa Ryan and Dr. Gill Diamond (UMDNJ-New Jersey Dental School) with permission of Dr. James M. Wilson (U. Pennsylvania)[18]. Six to 12 week old C57BL/6J (Jackson Laboratories, Bar Harbor, ME)r C3H/HeN (Harlan Laboratories) female mice were allowed to recover for at least 1 week following delivery. For inoculation, animals were anesthetized with inhaled isoflurane, and the urethra was catheterized as described[19]. 10^8^ or 5x10^5^ colony forming units (CFU) UPEC strain UTI89 were transurethrally introduced in 50 µl phosphate buffered saline (PBS). Once infection had progressed for 2, 6, 16, 24, or 48 hours, animals were re-anesthetized for sacrifice by cervical dislocation. 

### Bacterial burden - 10^8^ CFU inoculum

Bladders and kidneys were harvested into RPMI medium containing collagenase and DNase I at the time of sacrifice as described[20]. Organs were finely minced and agitated at 500 *g* for 30 minutes at 37°C. After filtering through 40 µm, homogenates were adjusted to 5 ml with sterile PBS. Serial log dilutions were plated on LB agar, and colonies were enumerated after 14 hour incubation at 37°C. Colony counts less than 9 were discarded, and the average colony counts for each sample were log-transformed. The detection threshold was 4500 CFU.

### Bacterial burden - 5x10^5^ CFU inoculum

Bladders and kidneys were harvested into 1x PBS as described[21]. Kidneys were homogenized with 2.0 mm zirconium oxide beads (ZROB20, Next Advance, Averill Park, NY) in 0.4 ml 1x PBS using a Bullet Blender Blue BBX24B Homogenizer (Next Advance) at the “8” setting for 2 minutes. Bladders were homogenized with 3.2 mm stainless steel beads (SSB32, Next Advance) in 0.5 ml 1x PBS at setting “8” for 3 minutes. Serial log dilutions of tissue homogenates were prepared, plated onto LB agar, and colonies were counted after 14 hr incubation. The detection threshold was 100 CFU. 

### RT-PCR

Bladders and kidneys were bisected with sterile scissors, snap frozen in liquid nitrogen, and stored at -80°C until mRNA isolation. Frozen tissue was pulverized in 1 ml Trizol reagent (Invitrogen, Carlsbad, CA) using a Polytron homogenizer. Next, RNA was extracted using the TRIzol® Plus RNA Purification System (Invitrogen) and eluted in 50 µl sterile water. Up to 3 µg of total RNA were reverse transcribed using random hexamer oligonucleotides in a 20 µl reaction volume (Verso cDNA Synthesis Kit, Thermo Scientific, Waltham, MA). After dilution to 60 µl with sterile water, 2.5 µl complementary (c)DNA was used as template in a quantitative (q)RT-PCR reaction. Duplicate PCR reactions were performed using 2x master mix (Fisher). VIC-MGB labeled *Gapdh*, FAM-MGB labeled *Defb1, Defb3* and *Defb14* primer/probe sets were used in separate reactions (Applied Biosystems, Carlsbad, CA). Alternatively, to measure Human *DEFB1* expression, duplicate PCR reactions were performed using 2x master mix containing Sybr Green (Fisher) and the following primers: *DEFB1* Forward 5’-TCA CTC CCA GCT CAC TTG CAG C-3’ and Reverse 5’-ATG GCC TCA GGT GGT AAC TTT CTC A-3’; *GAPDH* Forward 5’-GGT GGT CTC CTC TGA CTT CAA CA-3’ and Reverse 5’-GTT GCT GTA GCC AAA TTC GTT GT-3’[17]. PCR products were amplified and detected using the 7500 Real-time PCR System (Applied Biosystems). PCR threshold cycles (CT) were determined, and each cDNA was normalized for *GAPDH* (or *Gapdh*) content (ΔCT). For both human and mouse samples, relative expression changes were calculated using the 2^-ΔΔCT method, normalizing to a common pool of uninfected kidney or bladder cDNA[22]. 

### HBD-1 and mBD-14 immunohistochemistry

The distribution of HBD-1 protein within human ureter and bladder was evaluated as described[17]. Briefly, 4 µm sections were deparafinized, rehydrated, and subjected to antigen retrieval in a pressure cooker for 20 min using 10 mM sodium citrate buffer (pH 6.0). After blocking endogenous biotin (Biotin Block, ScytTek Laboratories, Logan, UT) and nonspecific protein (Superblock, ScyTek), slides were incubated at 4 °C overnight with polyclonal rabbit antibody against full-length BD-1 diluted 1:500 (sc-20797, Santa Cruz Biotechnology, Dallas, TX, affinity purified by the manufacturer) in PBS containing 3% fetal bovine serum. The presence of antibody-HBD-1 complexes was detected with biotinylated anti-polyvalent secondary antibody (ScyTek) and UltraTek Streptavidin/HRP (ScyTek). Sections were developed using 0.1% diaminobenzidine tetrahydrochloride (Arcos Organics, Geel, Belgium) with 0.01% hydrogen peroxide and counterstained with hematoxylin. The specificity for HBD-1 reactivity was confirmed through the use of unimmunized rabbit serum in place of HBD-1 antibody. The distribution of mouse BD-14 (mBD-14) protein was evaluated in a similar fashion, using affinity-purified anti-mBD14 raised in goat (diluted 1:100)[23] and detected using biotinylated anti-goat secondary antibody (Scytek). We attempted to localize mBD-1 within the bladder, but equivalent staining was observed in wild type and Defb1^-/-^ tissues over a wide range of antibody dilutions using the following commercially available primary antibodies directed against mBD-1: sc-25573 (rabbit polyclonal, Santa Cruz Biotechnology), sc-10851, (goat polyclonal, Santa Cruz Biotechnology), LS-C20902 (rabbit polyclonal, LifeSpan Biosciences, Seattle, WA), and MBD11-A (rabbit polyclonal, Alpha Diagnostic International, San Antonio, TX) indicating non-specificity of commercially available anti-murine antibodies. 

### Recombinant AMPs and bactericidal activity

Recombinant mBD-3 was synthesized in *E. coli* (5987-BD, R&D Systems, Minneapolis, MN). Recombinant mBD-14 was synthesized as a mature peptide in *E. coli* and purified as described[23]. Antimicrobial activity of recombinant peptides was evaluated by microdilution assay. Briefly, UTI89 bacteria (10^5^ CFU) were incubated with 0.3125, 0.625, 1.25, 2.5, 5, or 10 µM of mBD-3 or mBD-14 in 50 µl 0.1X PBS buffer for 3 hours at 37°C, then plated overnight on LB at 37°C. Next, the number of CFU at each concentration of peptide was determined. Using untreated bacteria as baseline, the minimum inhibition concentration (MIC) was identified by the lowest peptide concentration inhibiting growth of 90% of the inoculum as described previously[17]. Sensitivity of the UTI89 bacterial strain to killing by antimicrobial peptides was verified with the use of recombinant human RNase 7, yielding MIC of 0.2-0.4 µM, consistent with published data[24]. 

### Statistics

For comparing bacterial burden in wild type versus *Defb1* ^*-/-*^ mice, log-transformed CFU from kidneys and bladders were compared by the Mann-Whitney test (GraphPad Software, La Jolla, CA). For comparison of qRT-PCR results in infected versus uninfected tissues, an unpaired Student’s 2-tailed *t*-test assuming unequal variation was used. *P* values of < 0.05 were considered significant. 

## Results

**Figure 1:**
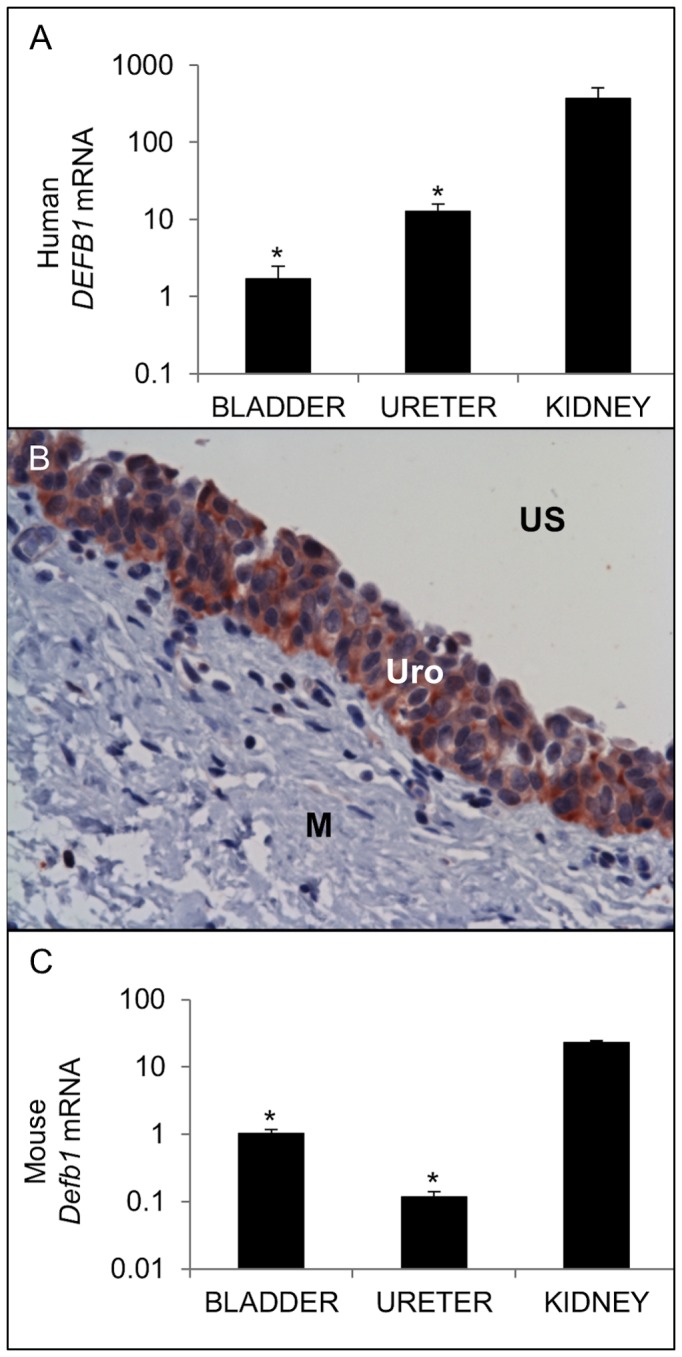
Expression of BD-1 in the uninfected urinary tract. (A) Expression of human *DEFB1* mRNA (TOP) and mouse *Defb1* mRNA (BOTTOM). Samples were normalized for *GAPDH / Gapdh* content and expressed as fold-difference compared to a pool of uninfected human / mouse bladder cDNA using the 2^-ΔΔCT method[22]. * indicates p < 0.05 in 2-tailed student’s *t*-tests comparing indicated organ to kidney. The average fold change ± standard error of the mean (S.E.M.) for each organ is shown (n=4 bladders, 2 ureters, 3 kidneys). (B) HBD-1 protein localizes to bladder urothelium by IHC. US: Urinary Space; Uro: Urothelium; M: Muscularis. Similar results were seen in ureter (data not shown). 400x original magnification.

We evaluated the expression of human *DEFB1* mRNA in kidneys, ureters, and bladders obtained from individuals without clinical or laboratory evidence of UTIs. *DEFB1* mRNA is detectable throughout the human urinary tract, albeit at significantly lower levels in bladder and ureter than those observed in the kidney (Figure **1A**). HBD-1 protein localized to all layers of the transitional urothelium of both ureter and bladder and was not detected in the submucosa or smooth muscle lining layer (Figure **1B**). 

We detected *Defb1* mRNA throughout the uninfected lower urinary tract of adult female mice, with significantly higher expression in kidneys than ureters and bladders (Figure **1C**). We attempted to evaluate mBD-1 protein expression by immunohistochemistry but encountered equivalent staining in wild type C57BL/6 and *Defb1* ^*-/-*^ tissues with all commercial antibodies tested, arguing against antibody specificity for this application.

**Figure 2:**
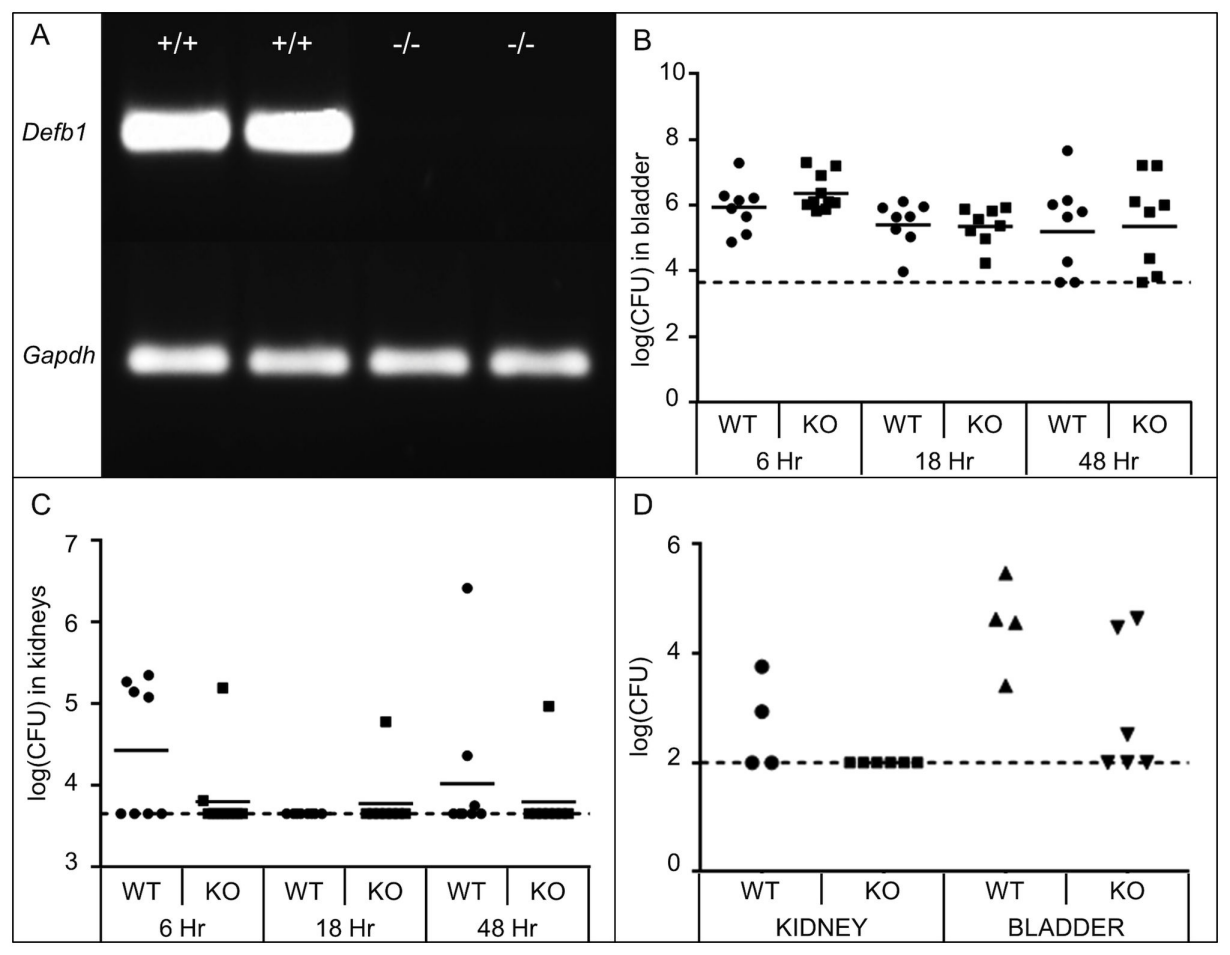
Effect of *Defb1* deficiency on UPEC burden. (A) RT-PCR confirms absent expression of *Defb1* mRNA in kidneys of *Defb1* ^*-/-*^ mice (-/-, n=2), versus presence of the predict PCR product in wild type kidneys (+/+). *Gapdh* RT-PCR is included as a loading control. (B) Bacterial burden in bladders and kidneys of wild type (WT) versus *Defb1* ^*-/-*^ (knockout, KO) mice at indicated timepoints following UPEC inoculation. Compiled data from at least 2 separate experiments in 8 mice are shown. The horizontal lines indicate geometric means. The dashed horizontal line indicates the lower limit of detection. There was no significant difference between geometric means at any time following infection in bladders or kidneys (Mann-Whitney Test, *p* > 0.05). (C) Bacterial burden in bladders and kidneys of wild type (WT) versus Defb1^-/-^ (knockout, KO) mice at 24 hpi following inoculation with 5x10^5^ CFU UTI89.

Since Defb1^-/-^ animals have a reportedly higher incidence of spontaneous bacteriuria[16], we hypothesized that the absence of *Defb1* would result in increased bacterial burden in bladders and kidneys following transurethral inoculation of UPEC strain UTI89 compared to age and strain matched wild type controls. We confirmed absent expression of *Defb1* mRNA in uninfected Defb1^-/-^ kidneys by RT-PCR (Figure **2A**). *Defb1* deficiency was not associated with significant changes in the quantity of live UPEC recovered from bladders and kidneys at 6, 16 and 48 hours post-inoculation (hpi) with 10^8^ CFU of UTI89 (Mann-Whitney test, *p* > 0.05; Figure **2B and 2C**). In these same experiments, UPEC clearance was observed in a greater proportion of Defb1^-/-^ than wild type kidneys at 6 hpi (Figure **2C**). Since AMP deficiency has been associated with increased susceptibility to lower UPEC inocula, we separately challenged Defb1^-/-^ and wild type control mice with 5x10^5^ CFU of UTI89[25]. At this lower inoculum, 100% (6/6) of Defb1^-/-^ animals demonstrated undetectable UPEC in their kidneys, compared to 50% (2/4) of wild type animals (Figure **2D**). 

**Figure 3:**
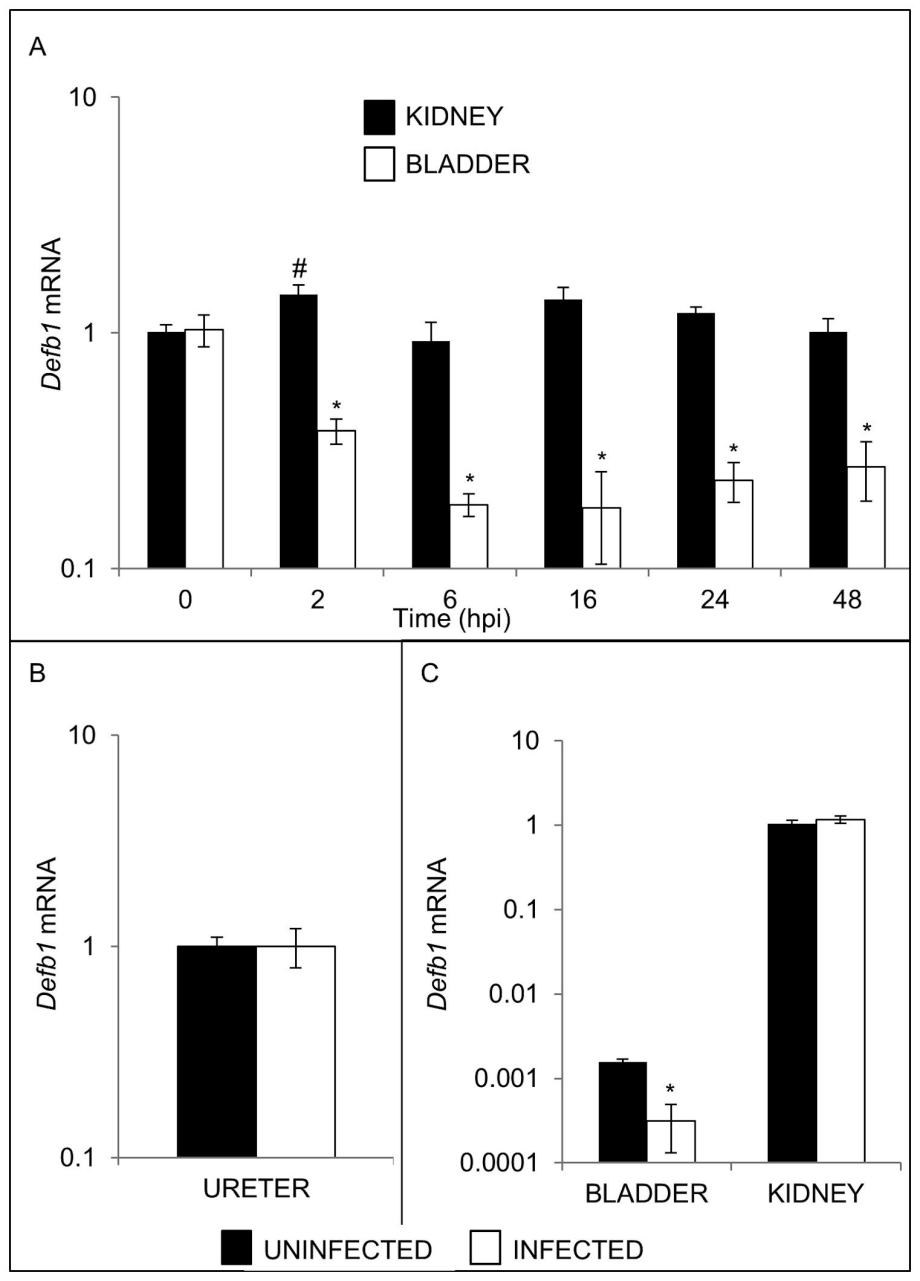
Effect of UPEC infection on *Defb1* mRNA expression. (A) Timecourse of *Defb1* mRNA expression in bladders (open boxes) versus kidneys (shaded boxes) of UPEC infected mice. Time in hpi is indicated on the X axis. Samples were normalized for *Gapdh* mRNA expression and compared to a pool of uninfected bladder or kidney cDNA using the 2^-ΔΔCT method[22]. Means ± S.E.M. are shown (n=4 bladders and 4 kidneys/group) * *p* < 0.05, student’s *t*-test, indicated timepoint versus uninfected bladders; # p < 0.05, student’s t-test, indicated timepoint versus uninfected kidneys. (B) *Defb1* mRNA was measured in ureters from uninfected C57BL/6 females (shaded box) and 16 hpi with UTI89 (open box). Samples were normalized for *Gapdh* content and expressed as fold-difference compared to a pool of uninfected mouse ureter cDNA using the 2^-ΔΔCT method. There was no significant difference in *Defb1* expression between groups. (C) Bladder *Defb1* mRNA expression is down-regulated in C3H/HeN mice 16 hpi, but kidney *Defb1* mRNA levels are unchanged. Shaded boxes represent uninfected organs, and open boxes represent organs harvested 16 hpi. Samples were normalized for *Gapdh* mRNA expression and compared to a pool of uninfected kidney cDNA using the 2^-ΔΔCT method[22]. Means ± S.E.M. are shown (n=3 bladders and 3 kidneys/group) * *p* < 0.05, student’s *t*-test, naïve versus infected C3H/HeN bladders.

Decreased expression of *Defb1* mRNA is a known immune escape mechanism utilized by pathogens outside of the urinary tract, and UPEC is known to actively suppress the production of cytokines produced by bladder epithelium[26–33] We therefore evaluated bladder and kidney expression of *Defb1* mRNA levels at 2, 6, 16, 24, and 48 hpi. UPEC significantly decreased bladder *Defb1* mRNA levels as early as 2 hpi, reaching a nadir by 6 hours (Figure **3A**). In contrast, no reduction in *Defb1* mRNA levels was observed in kidneys (Figure **3A**) or ureters (Figure **3B**). *Defb1* mRNA levels were also reduced to a comparable extent in the bladders of C3H/HeN mice inoculated with UPEC, but kidney levels were unaffected (Figure **3C**).

**Figure 4:**
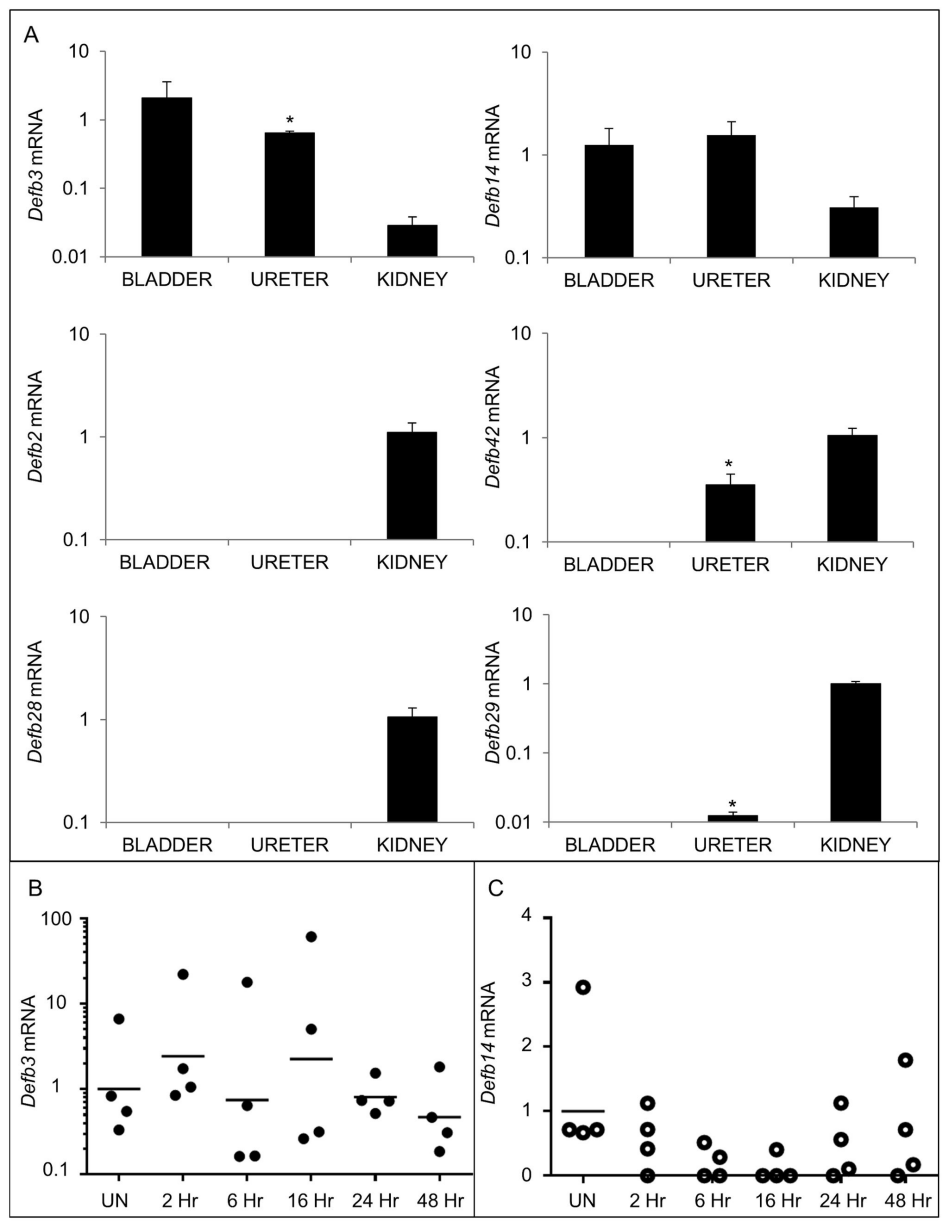
Additional BDs are expressed in the lower urinary tract. (A) Expression of *Defb* mRNAs in uninfected kidneys, ureters, and bladders. Samples were normalized for *Gapdh* content and compared to a pool of uninfected bladder cDNA using the 2^-ΔΔCT method[22]. Means ± S.E.M. are shown from 4 bladders, ureters, and kidneys. * indicates *p* < 0.05 in a 2-tailed Student’s *t*-test comparing the indicated organ to kidney. (B) *Defb3* and (C) *Defb14* mRNA expression in bladder following UPEC infection. Time in hpi is indicated on the X axis. All samples were normalized for *Gapdh* content and compared to a pool of uninfected bladder cDNA using the 2^-ΔΔCT method[22]. The horizontal line indicates the geometric mean for each timepoint. Samples on the X-axis had undetectable *Defb14* mRNA expression. (n=4 animals/group; Student’s 2-tailed *t*-test, *p* > 0.05 for all comparisons of uninfected versus infected timepoints).

**Figure 5:**
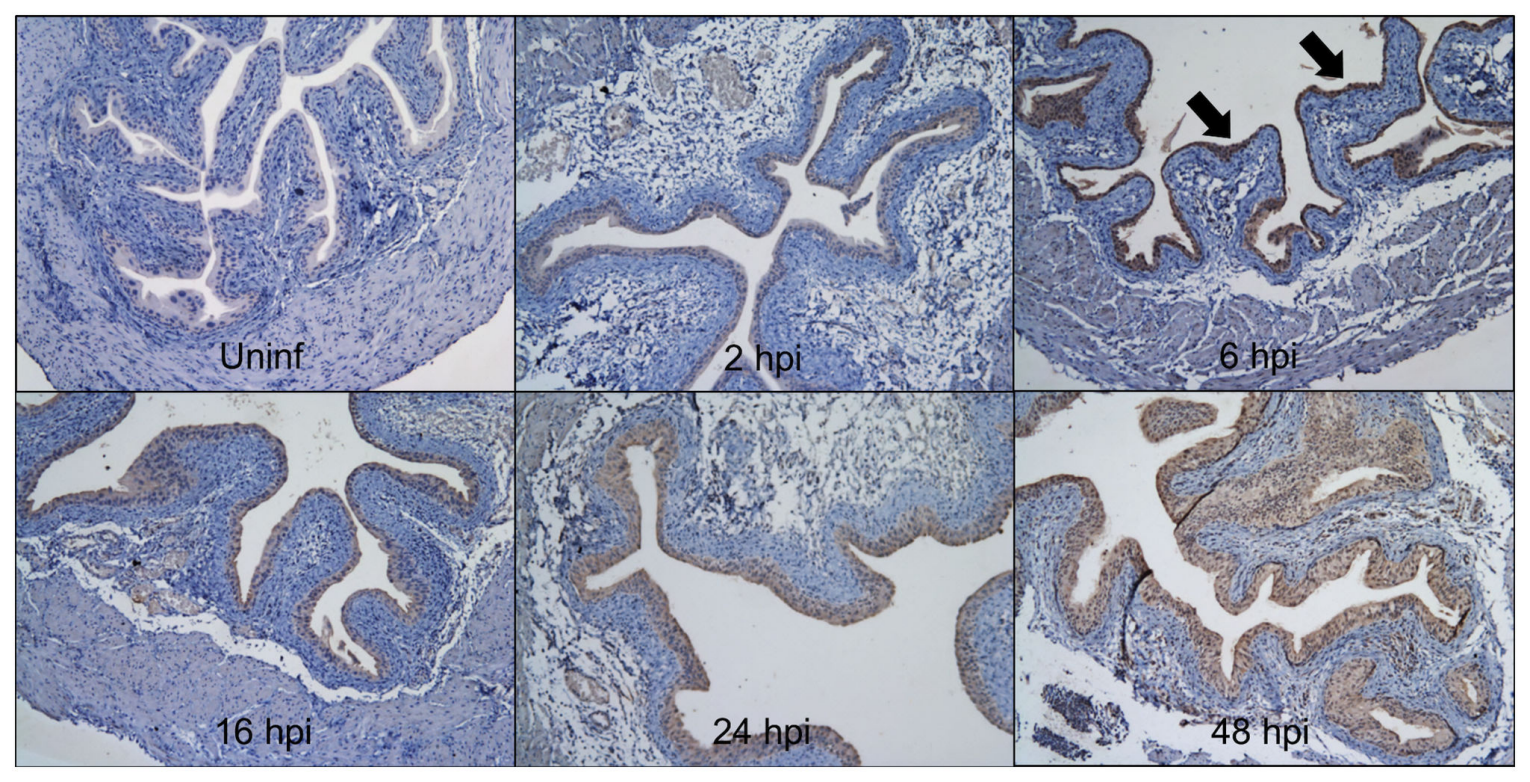
Bladder mBD-14 protein expression is induced by UPEC infection. Naïve and infected bladders harvested at indicated hpi were subject to mBD-14 IHC and counterstained with hematoxylin. Whereas mBD-14 is undetectable in uninfected bladder, it is expressed throughout the urothelium as early as 2 hpi, with increased expression by 6 hpi (black arrows) that persists at subsequent timepoints. 100x magnification.

Because *Defb1* deficiency did not significantly affect UPEC burden, we investigated whether additional β-defensins are expressed in the murine lower urinary tract. We determined the relative expression of *Defb* mRNAs within the urinary tract by qRT-PCR using cDNA from whole tissue homogenates of uninfected kidney, ureter, and bladder. Murine *Defb2* and *Defb28* mRNA were detected only in the kidney (Figure **4A**). *Defb29* and *Defb42* mRNA were undetectable in the bladder, but observed in the ureter and kidney. In contrast, *Defb3* and *Defb14* mRNA expression was detected in all three urinary tract tissues and selected for further analysis in the bladder (Figure **4A**). Variable *Defb3* mRNA levels were observed at baseline and throughout the 48 hour course of UPEC infection, but these differences did not reach statistical significance (Figure **4B**). *Defb14* mRNA levels were detectable in 100% (4/4) uninfected bladders but below the limit of detection in 75% (3/4) bladders at 16 hpi (Figure **4C**). IHC with a polyclonal antibody toward mBD-14 revealed undetectable expression in naïve murine bladder, low levels of expression in bladder urothelium within 2 hpi, and more intense staining throughout the urothelium by 6 hpi that persisted at 16, 24, and 48 hpi (Figure **5**). 

We next determined the potential susceptibility of UTI89 bacteria to the microbicidal activity of recombinant mBD-3 and mBD-14 *in vitro*. The MICs for mBD-3 and mBD-14 were 1.25-2.5 µM and 0.13-0.25 µM, respectively. 

## Discussion

[34]The innate immune system plays key roles in the detection and eradication of uropathogens, but the details of the complex interactions between host urothelium, leukocytes, and microbes remain incompletely elucidated[1]. Recent studies stress the importance of epithelial derived AMPs in maintaining sterility in the urinary tract[17,24,35–39]. For example, mice deficient in genes encoding AMPs such as cathelicidin-related antimicrobial peptide (*Cramp*) and Tamm-Horsfall protein (Thp) displayed greater susceptibility to UPEC than wild type controls[25,35,40]. In humans, when urinary Ribonuclease 7 (RNase 7) is neutralized *in vitro*, uropathogenic bacterial growth increases[17]. Deficiencies in these innate mucosal defenses may result in acute and/or chronic infection [6,37,39,41–43]. 

Our results demonstrate that murine *Defb1* and human *DEFB1* mRNA are abundantly expressed throughout the urinary tract and that the bladder urothelium produces HBD-1 peptide. These findings complement the work of Valore et al. who found that *DEFB1* mRNA and HBD-1 peptide are constitutively expressed by the epithelial lining of the nephron and secreted into the urinary stream[9]. Similarly, these findings parallel our research group’s work demonstrating that the renal collecting tubules and the urothelium of the lower urinary tract produces RNase 7[17]. However, while the kidneys and bladder secrete high concentrations of RNase 7 into the urinary stream sufficient to kill bacteria, urinary HBD-1 peptide expression is much lower[9,11,12]. Although urinary levels of HBD-1 are insufficient to kill invading bacteria, HBD-1 may provide a fast-acting antimicrobial coating of tubular lumens in the upper urinary tract to prevent infection by inhibiting bacterial attachment to the urothelium and serving as a second-line chemical shield[44].

In this study, our primary hypothesis was that *Defb1* deficiency results in increased susceptibility to UPEC UTI. This hypothesis was supported by published findings: (1) HBD-1 exhibits bactericidal activity toward UPEC *in vitro* [9]; and (2) Defb1^-/-^ mice display increased incidence of spontaneous bacteriuria[16]. We challenged *Defb1* ^*-/-*^ animals with UPEC because of the established nature of this gram-negative infection model in C57BL/6 mice and the predominance of UPEC among uropathogens in humans with UTI[1,19]. Interestingly, our experiments did not reveal any significant impact of *Defb1* deficiency on UPEC burden in bladders and kidneys of infected mice. This finding may be due to the choice of UPEC and/or mouse strains used in these experiments. Alternatively, mBD-1 may serve an important role in preventing spontaneous infection or clearance of low bacterial inocula, but may be dispensable for bacterial clearance when mice are challenged with large bacterial inocula. This has been previously observed in mice deficient in the *Thp* gene[25]. Whereas Thp^-/-^ and wild type controls had comparable bladder bacterial burden following inoculation with 10^8^ CFU UPEC, a greater proportion of Thp^-/-^ bladders was infected when the inoculum ranged from 10^4^ to 10^6^ CFU[25]. However, when we tested this hypothesis by infecting Defb1^-/-^ and wild type mice with a reduced inoculum, we did not observe any significant difference in bladder bacterial recovery. 

While *Defb1* deficiency did not affect bladder bacterial burden in experimental UTI caused by UPEC, we acknowledge that Defb1^-/-^ mice may exhibit greater susceptibility to gram-positive uropathogens than wild type controls. This hypothesis is supported by the observation that *Staphylococcus* species were predominantly isolated from urine of Defb1^-/-^ animals with spontaneous bacteriuria[16]. The experimental UTI described in this study were conducted in the C57BL/6 genetic background, which has been shown to rapidly clear *S. saprophyticus* from the bladder following transurethral inoculation[45]. It is conceivable that genetic deficiency of *Defb1* may confer susceptibility of C57BL/6 mice to *S. saprophyticus*. Future studies are required to test the hypothesis that *Defb1* deficiency confers susceptibility to gram-positive uropathogens, to elucidate whether *Defb1* expression is modulated by gram-positive infection in wild type mice, and to determine if mBD-3 and mBD-14 display antimicrobial activity toward gram-positive uropathogens *in vitro*. 

The observed similarities in UPEC burden recovered from Defb1^-/-^ and wild type animals may be due to functional diversification and/or redundancy among *Defb* genes within the lower urinary tract. This hypothesis is supported by our unexpected discovery that β-defensin expression in the naïve urinary tract is more complicated than previously suspected, with multiple *Defb* transcripts detectable in each organ and differential expression of *Defb* family members between the kidney, ureter and bladder. Detection of *Defb3* and *Defb14* mRNA in the naïve lower urinary tract led us to evaluate expression following experimental UTI and to explore the bactericidal activity of mBD-3 and mBD-14 toward UPEC. Indeed, we found that mBD-14 protein is detectable in UPEC infected bladder urothelium by IHC, and that mBD-3 and mBD-14 exhibit microbicidal activity toward UPEC in the low micromolar and nanomolar range, respectively. The MIC values measured in this study for mBD-3 and mBD-14 are comparable to those reported toward *E. coli* in the literature[46,47]. These findings lead us to hypothesize that *Defb3* and *Defb14* promote UPEC clearance *in vivo*. Future studies using mice with single, double, or triple deficiencies in *Defb1*, *Defb3*, and *Defb14* will ultimately demonstrate the relative contribution of these genes and their protein products to UPEC clearance and establish whether eradication of UPEC depends on a particular *Defb* gene or the coordinated, functionally redundant expression of multiple *Defb* genes.

Whereas *Defb1* is dispensable for UPEC clearance *in vivo*, we unexpectedly found that UPEC inhibits bladder expression of *Defb1* mRNA in wild type mice. This bladder-specific decrease in *Defb1* mRNA expression was observed at multiple times after UPEC inoculation and reproducible in both C57BL/6 and C3H/HeN mouse strains. The decrease in *Defb1* expression is not due to exfoliation of urothelium, as this was detectable 2 hpi when the urothelium was intact. The mechanisms responsible for UPEC regulation of *Defb1* mRNA expression are currently under further investigation. Since reduced *Defb1* mRNA levels are noted in lipopolysaccharide hyporesponsive C3H/HeJ mice in bladder urothelium 24 hpi with UPEC when compared to untreated or carrier-treated control bladders (http://www.ncbi.nlm.nih.gov/sites/GDSbrowser?acc=GDS2977), we hypothesize that intact Toll-like 4 receptor signaling may not be required for *Defb1* regulation[48]. Down-regulation of epithelial human *DEFB1* or murine *Defb1* mRNA expression has been demonstrated following bacterial and viral infection in the digestive and respiratory tract[30–33]. While the exact significance of *Defb1* mRNA down-regulation by UPEC remains unknown, this observation adds to the repertoire of host effector molecules such as interleukin (IL)-6 and IL-8 that are modulated by UPEC to subvert host innate immune responses[26,28,29]. 

While *Defb1* was dispensable for clearance of UPEC, we cannot exclude a direct role for *Defb1* in the host immune response to UPEC, which was not evaluated in this study. HBD-1 is expressed by a variety of immune cells including platelets, monocytes, and plasmacytoid dendritic cells[33,49]. HBD-1 triggers mast cell chemotaxis[50] and formation of neutrophil extracellular traps by polymorphonuclear leukocytes[49]. Whereas comparable expression patterns and activities have not yet been demonstrated with mBD-1, the other lower urinary tract BDs identified in this study also exhibit immunomodulatory activity outside of the urinary tract. Recombinant mBD-3 exhibits differential regulation of pro-inflammatory cytokines *in vivo* during murine influenza A infection, with upregulation of interferon-γ and interleukin (IL)-12 and downregulation of tumor necrosis factor (TNF)-α[51]. Recombinant mBD-14 has been implicated in stimulation as well as suppression of the innate immune response in macrophages and dendritic cells. Pretreatment of macrophages with mBD-14 results in increased Erk phosphorylation, CD86 and F4/80 expression, and production of TNF-α, IL-6, and CXCL2[52]. However, concomitant administration of mBD-14 with LPS results in down-regulation of TNF-α production by macrophages and serum TNF-α levels *in vivo*[53]. Thus, the temporal relationship between mBD-14 administration and microbial exposure appears to dictate whether the ensuing macrophage response is pro- or anti-inflammatory. Future studies should establish whether these observations apply during UPEC-mediated experimental UTI.

## Conclusions

In this study, we demonstrate for the first time that multiple BDs are expressed in the lower urinary tract, where they are subject to differential regulation by UPEC. This discovery mirrors published accounts demonstrating multiple β-defensin gene products in a variety of epithelial cells outside of the urinary tract[5,14,47]. The identification in this study of lower urinary tract *Defb3* and *Defb14* mRNA expression and demonstration of bactericidal activity of mBD-3 and mBD-14 toward UPEC leads us to hypothesize that these BDs may serve key roles in mucosal defense of the urinary tract. Furthermore, the overlapping expression pattern of *Defb3* and *Defb14* with *Defb1* raises the possibility of functional redundancy among BDs as a potential explanation for similar observations in *Defb1* ^*-/-*^ and wild type mice following UPEC challenge. Characterization of *Defb3* ^*-/-*^ and *Defb14* ^*-/-*^ mice following UPEC challenge – both individually and in combination with *Defb1* ^*-/-*^ animals - will ultimately define the contribution of these BDs to mucosal immunity toward UPEC *in vivo*. 
